# Synaptic Mitochondrial Respiration Differs Between the Prefrontal Cortex and Hippocampus in C57Bl/6NTac Mice

**DOI:** 10.1007/s12035-026-05961-2

**Published:** 2026-05-30

**Authors:** Gladys A. Shaw, Amy J. Wegener, Hannah Stadtler, Gretchen N. Neigh

**Affiliations:** Department of Neuroscience & Anatomy, Virginia Commonwealth University, 1101 East Marshall Street, Box 980709, Richmond, VA 23298 USA

**Keywords:** Mitochondria, Stress, Estrogen, Anxiety

## Abstract

We evaluated the impact of chronic repeated predation stress (CRPS) on open field behavior and synaptic mitochondrial respiration within the prefrontal cortex (PFC) and hippocampus (HPC). Male and female C57Bl/6NTac mice were subject to CRPS for 15 days during their adolescent (PND36-50) and early adult (PND57-71) stages. All animals were assessed for anxiety-like behavior in the open-field assay in adulthood. Brains were collected (PND106-108) and immediately used to assess synaptic mitochondrial respiration with the SeahorseXFe24 instrument. CRPS induced anxiety-like behavior in female mice in the open field, despite not observing stress effects on mitochondrial respiration within either sex. However, both sexes displayed significant region-specific differences in mitochondrial respiration. PFC mitochondria respiration was higher than synaptic mitochondrial respiration rates in the HPC across multiple mitochondrial dynamics. To further understand regional differences in mitochondrial respiration, we analyzed expression of estrogen receptor beta (*ESR2*) and uncoupling protein 2 (*UCP2*) as both are indicated in regulation of mitochondrial dynamics. In the HPC, females expressed higher levels of both genes compared to males. Further, hippocampal *ESR2* expression was elevated by CRPS in both sexes, suggesting a potential mechanism by which synaptic mitochondria are protected.

**Graphical Abstract:**

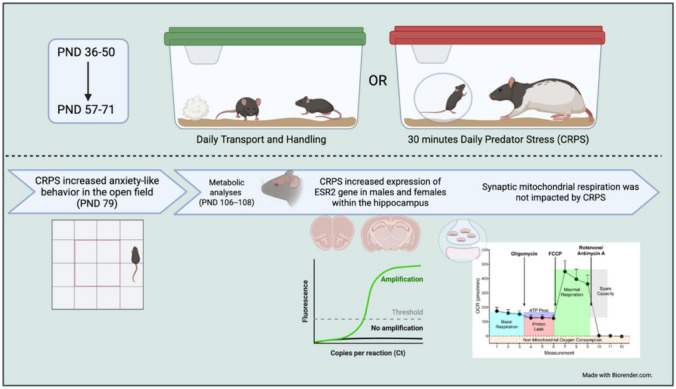

**Supplementary Information:**

The online version contains supplementary material available at 10.1007/s12035-026-05961-2.

## Introduction

Chronic stress has been shown to cause lasting neurobiological changes that can influence neuronal function [1]. For example, chronic stress has been shown to produce long-lasting impairments in cognitive performance and executive function that are associated with alterations in hippocampal (HPC) and prefrontal cortex (PFC) structure and function [2, 3]. However, the cellular and molecular mechanisms associated with these changes remain incompletely characterized.

Mitochondria have been implicated as potential mediators, as mitochondria are sensitive to glucocorticoids and other biological mechanisms associated with stress [4, 5]. Previous work has demonstrated that chronic stress produces persistent alterations in mitochondrial biology in both the periphery and the brain, including HPC mitochondrial “stress-response” transcriptional signatures and protein-level changes [6]. Additionally, studies using chronic stress paradigms in rodents have shown enduring changes in mitochondrial bioenergetics, including altered oxidative respiration measured from central mitochondria [7, 8].

Recently, our group has shown that chronic steady state exogenous corticosterone (CORT) administration in adult C57Bl/6 mice yields a treatment specific shift in mitochondrial respiration as measured from HPC synaptosomes [9]. However, the long-lasting effects of an endogenous stressor in adolescence, as well as the mechanisms involved in mitochondrial respiration within the PFC and HPC are still unknown. Furthermore, more work needs to be done to understand potential mechanisms of observed respiration differences. Estrogen receptor beta (encoded by *Esr2*) and uncoupling protein 2 (UCP2; encoded by *Ucp2*) are plausible modulators of these long-term bioenergetic effects. *Esr2* is highly expressed in HPC and PFC neurons, localizes to mitochondria, modulates oxidative phosphorylation and ROS balance, and has been implicated in stress responsivity and synaptic plasticity to a greater extent than estrogen receptor alpha [10, 11]. Similarly, *Ucp2* is enriched in neurons within stress-sensitive regions such as the HPC and PFC, regulates mitochondrial proton conductance, and has been implicated in synaptic plasticity and stress adaptation [12, 13].

Thus, this work aims to understand whether exposure to a two-hit model of predatory stress during adolescence and early adulthood results in persistent alterations in synaptic mitochondrial function in the PFC and HPC in adult mice. These data also examine the role of chronic stress in the induction of altered transcript level expression of *Esr2* and *Ucp2* within synaptic mitochondria, potentially mediating sex- and stress-differences in synaptic mitochondrial respiration within the PFC and HPC.

## Methods

### Animals

Forty postnatal day (PND) 22 male and female C57Bl/6NTac mice were purchased from Taconic Laboratories (*n* = 10 per group). All mice were placed in same sex pairs and given a single nestlet square for enrichment. The temperature and humidity-controlled colony room was kept on a 12:12 light:dark cycle, with lights in the animal housing facility turning on at 6 am and off at 6 pm. Mice had *ad libitum* access to both food and water in ventilated rack cages. The predators used in this study were 10 male Long Evans retired breeders (Charles River) who weighed 588 ± 14 g at the start of the study. Predator rats were food restricted to 85% of their starting body mass. Protocols used were approved by Virginia Commonwealth University’s Animal Care and Use committee.

### Chronic Repeated Predation Stress (CRPS)

Beginning on PND 36, mice were randomly assigned to undergo chronic repeated predatory stress (CRPS) and were isolate housed with no enrichment. Expanded methods are provided in the Supplemental Methods. Briefly, during CRPS sessions, mice were placed in a clean dwarf hamster ball to provide protection from physical harm but still allow odor exchange and interactions with the predator, including chewing and moving the ball around the cage. The ball was placed in the home cage of an adult male Long Evans retired breeders for 30 min daily for 2 weeks during adolescence (PND 36–50) and during adulthood (PND 57–71) [8, 14]

### Open Field

As a behavioral assessment of long-term effects of the CRPS paradigm, all mice were subject to 10 min in a grey open field arena (36 cm × 36 cm × 43 cm) under 700 lux lighting 3 to 5 h into the light cycle on PND 79. Open field testing took place during the light cycle in order to maintain consistency with the CRPS paradigm. Movement was tracked in real time using EthoVision XT 15.0 and an overhead camera at 25 frames per second. Center point of the animal was used to assess velocity, distance traveled, and location in the arena.

### Tissue Collection

Animals were euthanized via rapid decapitation. Mice were not placed under anesthesia, as anesthesia alters corticosterone levels in rodents [15]. Fresh tissue was collected at PND 106–108. The PFC and HPC from the right hemisphere were fresh dissected and immediately processed for synaptosomal isolation. PFC and HPC tissue from the left hemisphere were dissected and flash frozen for qPCR analysis of *Esr2* and *Ucp2* mRNA expression.

### Synaptosomal Mitochondria Isolation

Tissue was processed according to previous publications [8, 16, 17]. Using a 2 mL Dounce homogenizer, neural tissue was homogenized in 1.5 mL homogenizing buffer using 12 strokes of the plunger. Homogenate was spun at 3600×g for 10 min at 4 °C; 1.2 mL of supernatant was then gently added atop a 5-layer discontinuous, isotonic Percoll gradient composed of 1 mL of 23%, 15%, 10%, 3% Percoll, and 0.5 mL of 0% Percoll. Prepared gradients with homogenate were then spun at 32,000×g for 10 min at 4 °C in a Sorvall MX-120+ ultracentrifuge and a fixed-angle S58A rotor.

Synaptosomes were removed from the interface of the 10% to 15% Percoll layers and the 15% to 23% Percoll layers and placed in a clean ultracentrifuge tube with 6 mL of ionic media (isotonic solution; see [8, 14] for composition) and spun at 15,000×g for 35 min at 4 °C in a fixed angle S58A rotor. Synaptosomes, pelleted at the bottom of the tube, were collected and assessed for protein content via absorbance measurements via NanoDrop.

### Seahorse Cell Mito Stress Test

Isolated synaptosomes were diluted to 40 µg synaptosomes per 100µL of ionic media and plated in triplicate in poly-D lysine-coated Seahorse XFe24 cell plates (Agilent Technologies). Samples that did not yield 40 µg of synaptosomes were not included in the analysis. The prepared plates were spun at 3400×g for 30 min at 4 °C to allow adherence of synaptosomes. Plates were incubated in a non-CO_2_ incubator for 30 min before changing the ionic media for warmed Seahorse XF media. The cell mitochondrial stress test (Agilent Technologies, PN: 103015-100) was used to assess mitochondrial respiration, composed of Oligomycin A (2.0 µM), FCCP (1.0 µM), and Rotenone/Antimycin A (0.5 µM).

### RNA Extractions

Tissue was homogenized in 400 µL RLT buffer via the Tissue Lyser II instrument (Qiagen) using 1.4 mm ceramic beads (Omni International Cat # 19–645-3 Batch 2156390). Tissues were homogenized at 30/s for 45 s (HPC) or 3 min (PFC), followed by a 3-min spin through a QiaShreader. All RNA extractions were completed using the Qiagen Mini Kit and QiaCube instrument. RNA concentration and purity were assessed via Nanodrop (ThermoFisher). RNA was diluted to 0.25 µg/20 µL for cDNA conversion and converted using the AB High-Capacity cDNA reverse transcriptase kit according to manufacturer’s instructions.

### Quantitative Polymerase Chain Reaction

TaqMan quantitative polymerase chain reaction (qPCR) was completed on cDNA samples run in triplicate on a QuantStudio 6 instrument. Relative transcript levels of estrogen receptor ß (*Esr2*; ThermoFisher Scientific, Cat # Mm00599821_m1) and the mitochondrial uncoupling protein 2 (*Ucp2*, ThermoFisher Scientific, Cat # Mm00627599_m1) were assessed. *Gapdh* (Cat. # Mm99999915_g1), and *Actb* (Cat. # Mm01205647_g1) were used for normalization of each sample as reference genes. The non-stressed males were set as the control group for relative expression.

### Statistical Analysis

Data from all behavioral and molecular tests were analyzed using GraphPad Prism 9.2 for Windows (GraphPad La Jolla, CA). All data were analyzed prior to statistical testing for normality to confirm the use of subsequent parametric tests with the D’Agostino & Pearson test. Two-way ANOVAs with the factors of stress and sex were utilized for behavioral endpoints. Seahorse data were analyzed via three-way ANOVAs with fixed effects for region, stress, and sex. When a significant main or interaction effect was detected, post hoc Fisher’s LSD tests were utilized. Pearson’s correlations between OF metrics of distance traveled, velocity, percent time in center, or percent time in periphery and basal respiration for the PFC or HPC were examined across eight comparisons. *Esr2* and *Ucp2* expression were analyzed via two-way ANOVAs with fixed effects for sex and stress. An *α* = 0.05 was used for all statistical assessments and Bonferroni correction was employed for multiple comparisons.

## Results

### Open Field

**Fig. 1:**
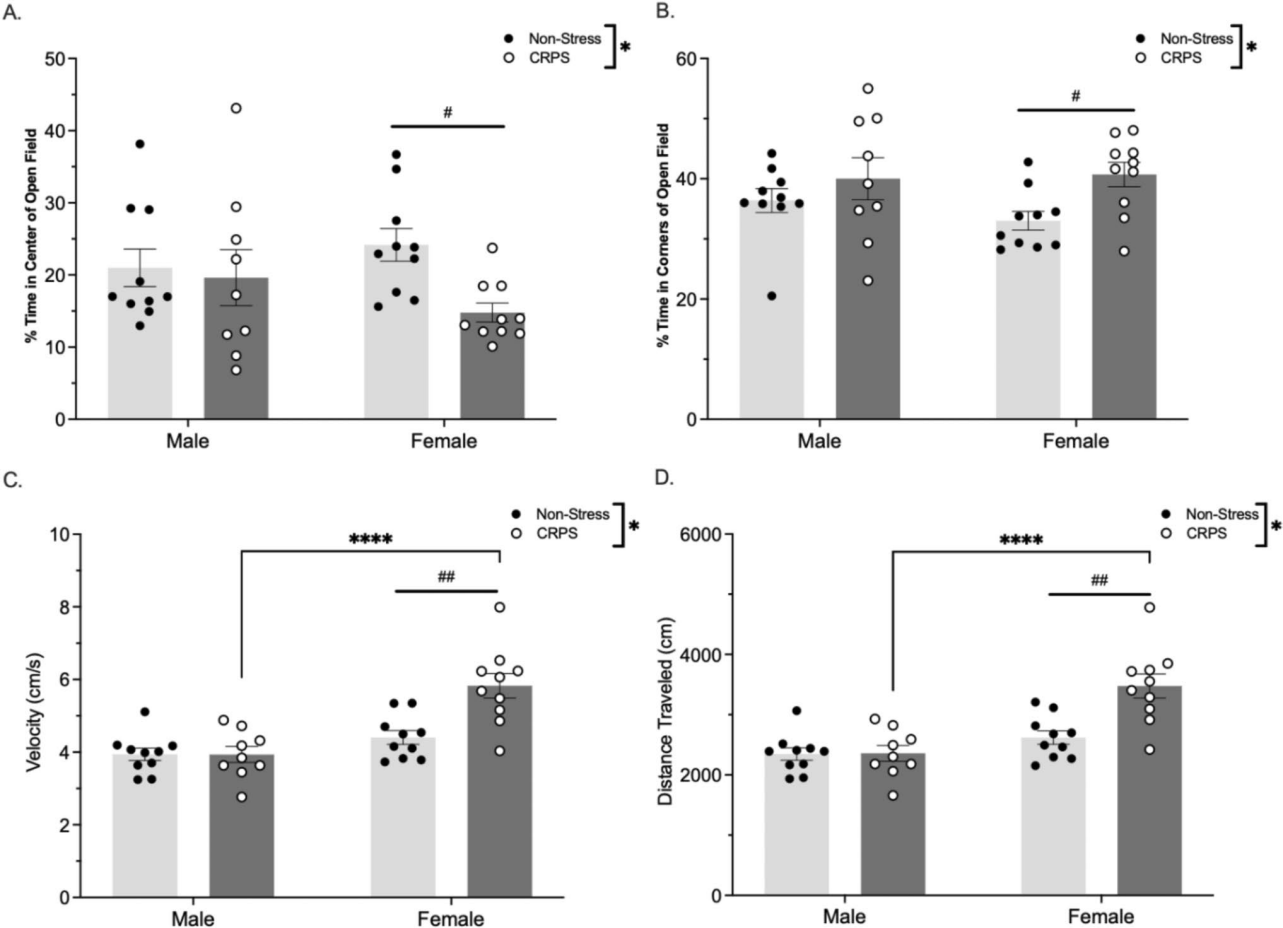
A history of CRPS induces anxiety-like behavior within the open field assay. Male mice with a history of CRPS (*n* = 9) and no stress (*n* = 10), and female mice with a history of CRPS (*n* = 10) and no stress (*n* = 10) were assessed in the open field arena for anxiety-like behavior. Sample sizes differed as one male mouse experienced physical injury during CRPS paradigm and was excluded. **A** There was an overall effect of stress, as mice with a history of stress display increased anxiety-like behavior as shown by decreased time spent in the center of the arena (*F* = 4.200, *p* = 0.047). **B** CRPS mice spent more time in the corners of the arena (*F* = 6.000, *p* = 0.019). In both **C** velocity and **D** distance traveled, there was an overall effect of stress (velocity: *F* = 8.700, *p* = 0.006; distance: *F* = 9.300, *p* = 0.004), sex (velocity: *F* = 24.000, *p* =  < 0.001; distance: *F* = 23.000, *p* < 0.001), and a significant interaction effect (velocity: *F* = 8.800, *p* = 0.005; distance: *F* = 8.700, *p* = 0.005). Females with a history of stress exhibited increased hyperactivity compared to non-stressed females and CRPS males. Bars represent mean ± SEM, *p < 0.05 of overall effect of stress, #*p* < 0.05 effect of stress, ##*p* < 0.01 effect of stress, ****p < 0.001 interaction effect

A history of stress-induced anxiety-like behavior within the open field. Stressed mice spent less time in the center of the arena (*F*_(1,35)_ = 4.246, *p* = 0.046; Fig. 1A) and more time in the corners of the open field arena (*F*_(1,35)_ = 6.004, *p* = 0.019; Fig. 1B) with a post hoc Fisher's LSD indicating CRPS females spent more time outside of the center (*t* = 9.369, p<0.014) in the periphery (t = -9.775, p = 0.009) compared to non-stressed females. The locomotor metric of velocity differed by sex (*F*_(1,35)_ = 24.06, *p* < 0.001), stress (*F*_(1,35)_ = 8.753, *p* = 0.006) and an interaction (*F*_(1,35)_ = 8.810, *p* = 0.005; Fig. 1C), such that females and CRPS mice displayed hyperactivity. A post hoc Fisher’s LSD revealed CRPS females displayed a higher velocity compared to non-stressed females (*t* = 4.249, *p* < 0.001) and CRPS males (*t* = 5.493, *p* < 0.001). Distance traveled was impacted by sex (*F*_(1,35)_ = 23.91, *p* < 0.001), stress (*F*_(1,35)_ = 9.312, *p* = 0.004), and an interaction between the variables (*F*_(1,35)_ = 8.782, *p* = 0.005; Fig. 1D). Again, a post hoc Fisher’s LSD indicated that CRPS females traveled further than non-stressed females (*t* = 4.312, *p* < 0.001) and CRPS males (*t* = 5.480, *p* < 0.001).

### Synaptosomal Respiration

**Fig. 2:**
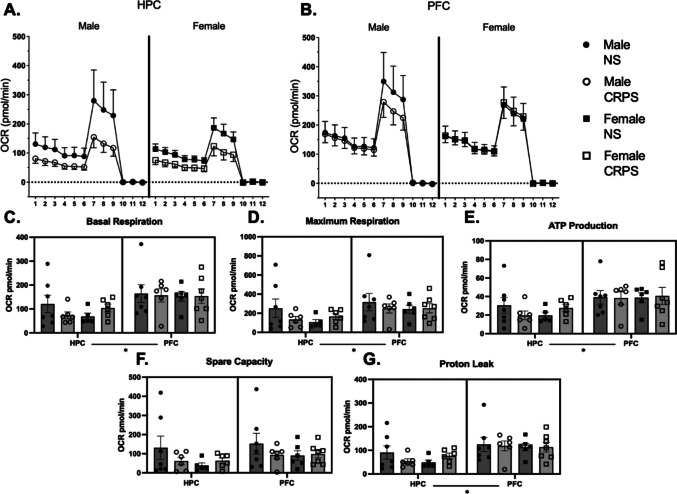
Chronic repeated predation stress does not impact synaptic mitochondrial respiration in the PFC or HPC. Hippocampus brain regions were isolated from male mice with a history of CRPS (*n* = 7) and no stress (*n* = 6), and female mice with a history of CRPS (*n* = 5) and no stress (*n* = 6). Prefrontal cortex was isolated from male mice with a history of CRPS (*n* = 7) and no stress (*n* = 6), and female mice with a history of CRPS (*n* = 6) and no stress (*n* = 7). Sample sizes differed based on insufficient protein yield, as samples with less than 40 µg of protein were excluded from testing. **A**, **B** Waveform representations of seahorse Cell Mito Stress test outputs. **C**–**G** There were no impacts of CRPS on male or female mice in any of the mitochondrial respiration metrics in the HPC or PFC. There was an overall effect of region for mitochondrial metrics of basal respiration, maximum respiration, ATP production, and proton leak, but no regional differences for spare capacity. For all mitochondrial metrics with a statistical difference, HPC exhibited lower respiration compared to the PFC. Bars represent mean ± SEM, *p < 0.05 effect of region

All groups started with *n* = 10. Following synaptosomal isolation (≥40 µg yield), group sample sizes were as follows: HPC—male CRPS (*n* = 7), male no stress (*n* = 6), female CRPS (*n* = 5), female no stress (*n* = 6); PFC—male CRPS (*n* = 7), male no stress (*n* = 6), female CRPS (*n* = 6), and female no stress (*n* = 7). Mitochondrial respiration metrics were analyzed by a three-way ANOVA on the basis of stress, sex, and brain region. There were no differences based on sex in any mitochondrial metric (basal respiration: *F*_(1,42)_ = 0.211, *p* = 0.648; maximum respiration: *F*_(1,42)_ = 1.084, *p* = 0.304; ATP production: *F*_(1,42)_ = 0.002, *p* = 0.957; proton leak: *F*_(1,42)_ = 0.338, *p* = 0.564; spare capacity: *F*_(1,42)_ = 2.062, *p* = 0.158). Similarly, there were no differences in any mitochondrial metric based on stress history (basal respiration: *F*_(1,42)_ = 0.052, *p* = 0.821; maximum respiration: *F*_(1,42)_ = 0.421, *p* = 0.519; ATP production: 0.009, *p* = 0.922; proton leak: *F*_(1,42)_ = 0.071, *p* = 0.792; spare capacity: *F*_(1,42)_ = 0.884, *p* = 0.353, Fig. 2).

However, there were overall significant region differences in mitochondrial metrics of basal respiration (*F*_(1,42)_ = 10.85, *p* = 0.002), maximum respiration (*F*_(1,42)_ = 5.105, *p* = 0.029), ATP production (*F*_(1,42)_ = 9.250, *p* = 0.004), and proton leak (*F*_(1,42)_ = 10.99, 0.002). Interestingly, there were no region differences based on mitochondrial spare capacity (*F*_(1,42)_ = 1.841, *p* = 0.182, Fig. 2).

There were no significant correlations among the open field metrics of distance traveled, velocity, percent time center, or percent time periphery and metrics of basal respiration for the PFC or HPC (all *p* > 0.00625 established by Bonferroni correction for multiple comparisons; see Supplemental Results for further details).

### qRT-PCR

**Fig. 3:**
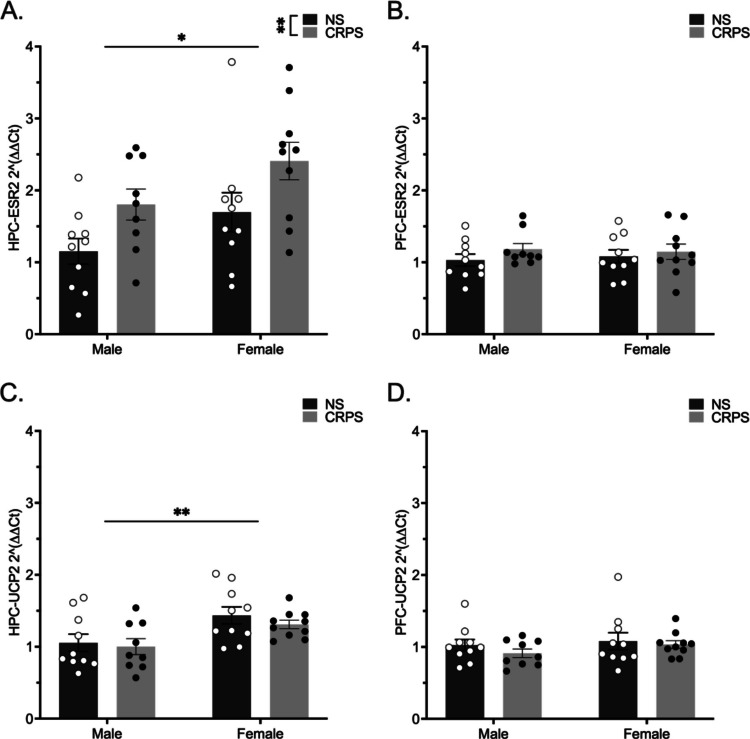
HPC *Esr2* gene expression is impacted by sex and stress. HPC *Ucp2* gene expression is impacted by sex. Male mice with a history of CRPS (*n* = 9) and no stress (*n* = 10), and female mice with a history of CRPS (*n* = 10) and no stress (*n *= 10) were assessed for *Esr2* and *Ucp2* mRNA expression in the HPC and PFC. **A** Females have higher HPC *Esr2* gene expression overall. HPC *Esr2* gene expression is increased in both males and females following a history of CRPS. **B** There are no sex or stress-induced differences in *Esr2* expression in the PFC. **C** Females express higher HPC *Ucp2* gene expression overall. **D** *Ucp2* expression in the PFC is not impacted by sex or stress. Bars represent mean ± SEM, *p < 0.05, **p < 0.01

Transcript levels of *Esr2*, the gene transcribing ERβ, were assessed using a two-way ANOVA for the factors sex and stress. A history of CRPS increased *Esr2* hippocampal expression in both males and females (*F*_(1,35)_ = 8.324, *p* = 0.007; Fig. 3A). Sex impacted hippocampal *Esr2* expression such that females expressed higher levels of *Esr2* compared to males (*F*_(1,35)_ = 5.922, *p* = 0.020). *Ucp2*, the gene transcribing the mitochondrial uncoupling protein Ucp2, was not impacted by CRPS in the hippocampus (*F*_(1,35)_ = 0.741, *p* > 0.05; Fig. 3C) but was higher in females regardless of stress history (*F*_(1,35)_ = 10.79, *p* = 0.002). Expression of *Esr2* in the PFC was not altered by stress (*F*_(1,35)_ = 1.420, *p* > 0.05; Fig. 3B) or sex (*F*_(1,35)_ = 0.007, *p* > 0.05). Similarly, *Ucp2* in the PFC was not altered by stress (*F*_(1,35)_ = 0.915, *p* > 0.05; Fig. 3D) or sex (*F*_(1,35)_ = 1.164, *p* > 0.05).

## Discussion

This work investigated the mitochondrial impacts of a two-hit model of CRPS in male and female mice. Although we observed behavioral effects of the paradigm including increased locomotor activity and decreased time in the center, consistent with previous studies using CRPS [8, 14], we did not find any differences in mitochondrial respiration on the basis of either sex or stress. Furthermore, there were no significant correlations between open field metrics and basal mitochondrial respiration in the HPC or PFC. However, both sexes demonstrate regional differences in mitochondrial respiration between the PFC and the HPC, with PFC synaptosomes having increased respiration at baseline, increased maximum respiration, increased proton leak, and increased ATP production compared to synaptosome mitochondrial from the HPC. There were no regional differences in mitochondrial spare capacity. Additionally, we found an increase in expression of *Esr2* in the HPC of male and female mice following CRPS, and a greater expression of *Ucp2* in the HPC of female, compared to male mice, regardless of stress history, suggesting potential compensatory roles of each protein in the HPC specifically.

We have previously demonstrated that chronic exogenous corticosterone lowers synaptic mitochondrial respiration in the hippocampus of both male and female mice [9]. We postulated that a CRPS paradigm would elicit similar impacts on synaptic mitochondria, but these effects were not observed in the current study. Directionality of stress modulation on synaptic mitochondrial respiration may be attributed to the type of stressor. Other studies have demonstrated chronic mild stress triggered mitochondrial respiratory inefficiency in cortical and hippocampal mitochondria not specific to the synapse [18]. Similarly, mitochondrial respiration in PFC punches is lowered in male rats following exposure to predator odor, but not females [19]. However, chronic restraint stress elevates complex I–III activity in both male and female rats in the hippocampus [20]. These studies support the role of mitochondria in preclinical stress paradigms. As such, the lack of effect of CRPS on synaptic respiration does not definitively indicate that synaptic mitochondria in the current study were unresponsive to the paradigm, as we cannot rule out adaptations by other mitochondrial metrics not assessed here.

Despite not observing an impact of CRPS on mitochondrial respiration in the current study, it could be beneficial in future studies to assess changes in morphological phenotype of these synaptic mitochondria. Previous data has shown that CRPS can influence synaptic mitochondrial morphology as indicated by the Flameng scoring system of mitochondrial health [8, 21]. In the aforementioned study, CRPS females had more synaptic mitochondria exhibiting a score of “4” indicating agranular and significantly damaged mitochondria. Such scorings suggest that CRPS females displayed more damaged mitochondria at the time of isolation while males did not have a visible change in mitochondrial morphology in response to stress. Further, this outcome was not reflected in CRPS female mitochondria respiration data. As such, the absence of synaptic respiration shifts does not definitively rule out the possibility that mitochondria were responsive to the stressor and requires future studies to fully characterize mitochondria morphology.

Independent of stress history, we did observe a regional difference between the HPC and PFC. The HPC and PFC are implicated in stress disorders but likely exhibit distinct metabolic consequences following stress specific to regional energetic requirements and resource availability for baseline function. In all mice, the PFC exhibited increased synaptic mitochondrial respiration compared to the HPC across basal respiration, maximum respiration, proton leak, and ATP production, but we did not identify differences in mitochondrial spare capacity on the basis of region. However, more work needs to be done to understand mechanistic underpinnings of different respiration by region. Regional differences could be influenced by mitochondrial number, localization, complex protein expression, or functionality and mutations [22]. The regional differences in mitochondrial dynamics could also be influenced by differing substrate-level availability or necessity in different brain regions. For example, a previous mouse study determined that the HPC and cortex exhibit regional differences in substrate-pool availability under basal conditions; however, this study did not specify the sex of the mice analyzed [23].

In order to tease apart the observed regional differences in the current study and to further examine mitochondrial impacts of the CRPS paradigm, we examined the expression of the *Esr2* and *Ucp2* that have established roles in modulating metabolic activity. Estrogens are known regulators of metabolic pathways [24, 25] and ERβ is especially relevant to substrate regulation in the setting of chronic stress [26]. Additionally, UCP2 is an important protein to mitochondrial health, as it acts to uncouple ATP production from the influx of protons between the inner membrane space and matrix of the mitochondria. This acts to increase the survival of neurons during periods of physiological stress [27]. Moreover, *Ucp2* expression is neuroprotective as it decreases reactive oxygen species production and Ca^2+^ influx within mitochondria, preventing activation of an apoptotic cascade [27–29]. We observed elevated expression of both genes in the HPC of female mice, relative to males, an effect not observed in the PFC. ATPase activity is directly modulated through ERβ [24, 30–33], acting as an activator of ATP production *in vitro* [33]. Additionally, *Esr2* was elevated in both males and females in the HPC following CRPS. Given the neuroprotective nature of estrogen, we postulate that *Esr2* expression is elevated in the CRPS animals to protect against the negative impacts of stress that have been reported in previous mitochondrial studies and consider this an important area of further inquiry.

There were limitations to this work. Importantly, measures of anxiety-like behavior were conducted on PND79, and tissue collection did not occur until PND 106—108. Discrepancies between anxiety-like behavior outcomes and mitochondrial outcomes could be due to the increased amount of time between the open field assessments and mitochondrial assessments. There was also an upregulation of *Esr2* expression in the HPC following CRPS that may have elicited protective qualities in the HPC across CRPS animals that prevented a stress-induced shift in synaptic mitochondrial respiration. Given the open field is not a hippocampal-associated task, additional behavioral studies that evaluate performance on hippocampal-associated memory tasks following CRPS and an estrogen receptor-targeted intervention may provide insight on the combined impact of *Esr2* and CRPS within this model. Furthermore, we did not analyze a brain region not expected to be influenced by manipulation, such as the occipital cortex, as a control measure. Lastly, although previous work has measured corticosterone concentration from mice that underwent CRPS [8, 9, 14], and ERβ signaling has been shown to modulate glucocorticoid-dependent neuroendocrine effects [26], CORT was not measured in this study. We are unable to make conclusions based on relationships between region-specific mitochondrial respiration, CORT, and *Esr2* expression. This study should be expanded into future work investigating the additional role of CORT within these relationships.

Overall, this study demonstrates that CRPS produces anxiety-like behavior and hyperactivity, particularly in females, while HPC and PFC mitochondrial respiration remains largely resilient to stress exposure. Instead, mitochondrial respiration measures reflected stable, region-specific differences. In parallel, CRPS increased hippocampal *Esr2* expression in both sexes, with females exhibiting higher *Esr2* and *Ucp2* expression overall, suggesting that stress-induced alterations in estrogen receptor signaling may occur independently from changes in synaptosomal mitochondrial respiratory function.

## Supplementary Information

Below is the link to the electronic supplementary material.

ESM1 (DOCX 17.8 KB)

## Data Availability

The datasets generated during the current study are not publicly available but are available from the corresponding author on reasonable request.
