# Supplementary material for: Synaptic Mitochondrial Respiration Differs Between the Prefrontal Cortex and Hippocampus in C57Bl/6NTac Mice
Source: Mol Neurobiol. 2026 May 30;63(1):663. doi: 10.1007/s12035-026-05961-2 (PMC13222168; doi:10.1007/s12035-026-05961-2)
Supplement: Supplementary file 1 — (DOCX 17.8 KB) [file 12035_2026_5961_MOESM1_ESM.docx]

**Supplemental Methods: Chronic Repeated Predation Stress (CRPS)**

**Materials & Animals:**

- Hamster balls (clear and green, 5”, Lee’s Aquarium & Pet Products, Catalog Number 20193 (colored) 20198 (clear) )
- Long Evans male rats (Charles River, ordered as Retired Breeders; starting body mass mean = 588 ± 14 grams; body mass was monitored weekly)

**Procedure:**

1. Prior to first day of stress paradigm all Long Evans used were individually housed. Long Evans rats were weighed and 85% of body weight was calculated as goal for food restriction. Food restriction was utilized to increase behavioral activation of the rats when used in the stress paradigm. Food restriction consisted of daily feeding of 16.8 grams of chow (Inotiv Teklad LM-485 Sterilizable Mouse/Rat Diet Cat# 7012). Weights were monitored weekly to ensure that rats did not drop below 85% of their starting body mass.

2. Control animals were transported to the testing room PRIOR to the stress procedures, handled briefly, left in the testing room for 30 minutes within their home cages, and then returned to the colony prior to the mice in the stress group or the Long Evans rats being brought to the room.

3. Mice in the stress group and Long Evans rats were transported to the testing room on each day of stress.

4. Each mouse in the stress group was placed into an individual hamster ball. Each ball was secured with tape in a cross-shaped pattern to protect against a rat successfully opening the hamster ball during the stress procedure.

5. One hamster ball containing one mouse was placed within the home cage of one Long Evans rat.

6. Pairings of each mouse-rat dyad were tracked and rotated such that each mouse was paired with each rat over the course of the days of the stress paradigm.

7. The interaction proceeded for 30 minutes with supervision to guard against any adverse events.

8. Behaviors were observed and qualitatively noted in the laboratory notebook but not quantified for each individual interaction. Long Evans rat behaviors included chewing on the tape and/or ball, rolling, pressing, and pushing the ball, sniffing at the ball, digging and kicking bedding around and under the ball. Mouse behaviors while within the hamster ball included self-rolling of ball, tail rattling, freezing, vocalizations, defecation, and urination.

9. At the end of 30 minutes, mice were released back into their home cage and remained in testing room for an additional 30 minutes prior to transport back to the colony. Rats remained in their home cages in the testing room during this time and were transported back to their colony room after the stress group mice were returned to their colony room.

10. This procedure was repeated daily during the stress paradigm.

**Supplemental Results**

In order to determine whether there were potential associations between behavioral metrics in the open field and basal respiration in synaptosome mitochondria from either the prefrontal cortex (PFC) or hippocampus (HPC), a series of Pearson’s correlations were assessed. Given that there were neither main effects of sex or stress exposure on basal respiration, correlations were conducted for the entire data set for each endpoint. In total, eight comparisons were assessed and are depicted in Supplemental Table 1. Bonferroni correction was used and the original α = 0.05 was adjusted to α = 0.00625 to control for the use of eight comparisons. After correction, no comparisons were significantly associated.

|  | **Percent Time in Center** | **Time in Periphery** | **Velocity** | **Distance Traveled** |
| --- | --- | --- | --- | --- |
| **PFC Basal Respiration** | (r(24) = 0.07, p = 0.7419) | r(24) = −0.065  p = 0.7507 | (r(24) = -0.42, p = 0.03069)  padj = 0.2456 | r(24) = 0.069  p = 0.7368 |
| **HPC Basal Respiration** | r(22) = -0.05, p = 0.8141 | r(22) = 0.046  p = 0.8301 | r(22) = 0.133, p = 0.8952 | r(22) = −0.118  p = 0.5841 |

**Supplemental Table 1.** Pearson correlations for outcome from the open field and basal respiration from synaptosome mitochondria from the prefrontal cortex (PFC) and hippocampus (HPC).
